# Evaluation of Neighborhood-Level Disadvantage and Cognition in Mexican American and Non-Hispanic White Adults 50 Years and Older in the US

**DOI:** 10.1001/jamanetworkopen.2023.25325

**Published:** 2023-08-30

**Authors:** Christina G. Wong, Justin B. Miller, Fan Zhang, Robert A. Rissman, Rema Raman, James R. Hall, Melissa Petersen, Kristine Yaffe, Amy J. Kind, Sid E. O’Bryant

**Affiliations:** 1 Cleveland Clinic Lou Ruvo Center for Brain Health, Las Vegas, Nevada; 2 Department of Neurosciences, University of California, San Diego, La Jolla; 3 Veterans Affairs San Diego Healthcare System, San Diego, California; 4 Alzheimer’s Therapeutic Research Institute, University of Southern California, San Diego; 5 Institute for Translational Research, University of North Texas Health Science Center, Fort Worth; 6 Department of Family Medicine, University of North Texas Health Science Center, Fort Worth; 7 Department of Psychiatry, Neurology, and Epidemiology and Biostatistics, University of California, San Francisco; 8 San Francisco VA Medical Center, San Francisco, California; 9 Center for Health Disparities Research, University of Wisconsin School of Medicine and Public Health, Madison; 10 Division of Geriatrics and Gerontology, Department of Medicine, University of Wisconsin School of Medicine and Public Health, Madison

## Abstract

**Importance:**

Understanding how socioeconomic factors are associated with cognitive aging is important for addressing health disparities in Alzheimer disease.

**Objective:**

To examine the association of neighborhood disadvantage with cognition among a multiethnic cohort of older adults.

**Design, Setting, and Participants:**

In this cross-sectional study, data were collected between September 1, 2017, and May 31, 2022. Participants were from the Health and Aging Brain Study–Health Disparities, which is a community-based single-center study in the Dallas/Fort Worth area of Texas. A total of 1614 Mexican American and non-Hispanic White adults 50 years and older were included.

**Exposure:**

Neighborhood disadvantage for participants’ current residence was measured by the validated Area Deprivation Index (ADI); ADI Texas state deciles were converted to quintiles, with quintile 1 representing the least disadvantaged area and quintile 5 the most disadvantaged area. Covariates included age, sex, and educational level.

**Main Outcomes and Measures:**

Performance on cognitive tests assessing memory, language, attention, processing speed, and executive functioning; measures included the Spanish-English Verbal Learning Test (SEVLT) Learning and Delayed Recall subscales; Wechsler Memory Scale, third edition (WMS-III) Digit Span Forward, Digit Span Backward, and Logical Memory 1 and 2 subscales; Trail Making Test (TMT) parts A and B; Digit Symbol Substitution Test (DSST); Letter Fluency; and Animal Naming. Raw scores were used for analyses. Associations between neighborhood disadvantage and neuropsychological performance were examined via demographically adjusted linear regression models stratified by ethnic group.

**Results:**

Among 1614 older adults (mean [SD] age, 66.3 [8.7] years; 980 women [60.7%]), 853 were Mexican American (mean [SD] age, 63.9 [7.9] years; 566 women [66.4%]), and 761 were non-Hispanic White (mean [SD] age, 69.1 [8.7] years; 414 women [54.4%]). Older Mexican American adults were more likely to reside in the most disadvantaged areas (ADI quintiles 3-5), with 280 individuals (32.8%) living in ADI quintile 5, whereas a large proportion of older non-Hispanic White adults resided in ADI quintile 1 (296 individuals [38.9%]). Mexican American individuals living in more disadvantaged areas had worse performance than those living in ADI quintile 1 on 7 of 11 cognitive tests, including SEVLT Learning (ADI quintile 5: β = −2.50; 95% CI, −4.46 to –0.54), SEVLT Delayed Recall (eg, ADI quintile 3: β = −1.11; 95% CI, −1.97 to –0.24), WMS-III Digit Span Forward (eg, ADI quintile 4: β = −1.14; 95% CI, −1.60 to –0.67), TMT part A (ADI quintile 5: β = 7.85; 95% CI, 1.28-14.42), TMT part B (eg, ADI quintile 5: β = 31.5; 95% CI, 12.16-51.35), Letter Fluency (ADI quintile 4: β = −2.91; 95% CI, −5.39 to −0.43), and DSST (eg, ADI quintile 5: β = −4.45; 95% CI, −6.77 to –2.14). In contrast, only non-Hispanic White individuals living in ADI quintile 4 had worse performance than those living in ADI quintile 1 on 4 of 11 cognitive tests, including SEVLT Learning (β = −2.35; 95% CI, −4.40 to –0.30), SEVLT Delayed Recall (β = −0.95; 95% CI, −1.73 to –0.17), TMT part B (β = 15.95; 95% CI, 2.47-29.44), and DSST (β = −3.96; 95% CI, −6.49 to –1.43).

**Conclusions and Relevance:**

In this cross-sectional study, aging in a disadvantaged area was associated with worse cognitive functioning, particularly for older Mexican American adults. Future studies examining the implications of exposure to neighborhood disadvantage across the life span will be important for improving cognitive outcomes in diverse populations.

## Key Points

**Question:**

Is neighborhood disadvantage associated with cognitive performance among older Mexican American and non-Hispanic White adults?

**Findings:**

In this cross-sectional study of 1614 participants (853 Mexican American and 761 non-Hispanic White) 50 years and older from the Health and Aging Brain Study–Health Disparities, living in a more disadvantaged neighborhood was associated with worse performance across several cognitive domains among Mexican American adults and worse performance on aspects of memory and processing speed among non-Hispanic White adults.

**Meaning:**

These findings suggest that understanding and addressing contextual social determinants of health associated with cognition will be important for improving aging outcomes, particularly among racial and ethnic minority populations.

## Teaser

This cross-sectional study examines the association between neighborhood disadvantage and cognition among Mexican American vs non-Hispanic White adults 50 years and older in the US.

## Introduction

Alzheimer disease (AD) disproportionately impacts older African American and Hispanic adults in the US.^1^ Older African American individuals currently experience the highest burden of AD, while older Hispanic individuals (65% of whom identify as Mexican American)^2^ are estimated to have the greatest increase in AD by 2060.^1^ Despite cognitive health disparities and projections of increasing racial and ethnic diversity in the US, Hispanic individuals are underrepresented in AD research.^3^ To develop advancements in AD prevention and treatment among diverse populations, this gap must be addressed.

The fundamental causes theory proposes that effective interventions for reducing health disparities require understanding of the distal (ie, fundamental) forces, such as socioeconomic context, that put people at “risk for (more proximal) risks,”^4^ such as individual-level income, educational level, and health behaviors. Previous research has found that these individual factors are associated with AD risk and play a role in health disparities in dementia prevalence.^5^ Although there is a growing body of literature pertaining to contextual-level social determinants of health, such as neighborhood disadvantage (ND), which is associated with structural and systematic inequities,^6,7^ less is known about the association of these factors with brain aging, especially for those with diverse backgrounds.

Living in a socioeconomically disadvantaged neighborhood increases the risk of poor health outcomes.^8,9,10,11^ For example, greater ND has been associated with increased cardiovascular disease–specific morbidity and mortality,^12,13^ and these increases are more pronounced for those with minority racial and ethnic backgrounds, such as African American individuals compared with non-Hispanic White individuals,^14,15^ suggesting that ND may be a key factor in health disparities. Greater ND may similarly confer increased risk of AD, especially among diverse communities.^16^

One validated measure of ND is the Area Deprivation Index (ADI), which uses 17 US Census and American Community Survey indicators of poverty, education, housing, and employment to characterize the level of disadvantage within Census block groups.^10,17,18^ The ADI has been associated with several AD-relevant factors by Kind et al.^17,18,19,20,21^ Examining cognitively unimpaired participants in the Wisconsin Registry for Alzheimer’s Prevention Study, Hunt et al^19^ found that living in the most disadvantaged neighborhoods was associated with greater cognitive decline and cortical thinning in AD signature regions. Moreover, these results remained significant for all but 1 region after accounting for racial and demographic differences between areas of high and low disadvantage.^19^ The same group also found lower hippocampal and total brain volumes among participants living in the most disadvantaged neighborhoods, even after accounting for differences in individual-level socioeconomic status.^20^ Furthermore, residing in areas of higher ND has been associated with increased odds of AD neuropathology.^21^ Other studies^22,23^ have confirmed these findings.

Of the limited studies examining ND and cognitive outcomes, few have focused on diverse populations.^3^ Some evidence suggests the association of neighborhood context with cognition may vary by race.^24,25^ For example, Rosso et al^25^ found greater differences in baseline cognitive performance between neighborhoods with high and low socioeconomic status for African American individuals compared with non-Hispanic White individuals. One study^26^ found that ND was associated with cognitive decline among older Mexican American adults, although other studies^25,27^ have not found a longitudinal association between neighborhood context and cognition. Notably, these studies^24,25,26,27^ examining neighborhood context and cognition in diverse cohorts used their own individually developed measures to capture ND, which may explain some of the discrepancies between studies. Methodological differences aside, accumulating evidence suggests that neighborhood context can play a role in cognition and that these cognitive outcomes may differ depending on race and ethnicity.

Due to legacies of structural inequalities, Hispanic individuals disproportionately reside in high-poverty US neighborhoods,^28^ which may be an important factor underlying the disproportionate increase in AD projected for the Hispanic American population. The primary aim of this study was to further our understanding of the association between ND and cognitive functioning among older Mexican American adults compared with older non-Hispanic White adults. We hypothesized that not only would greater ND be associated with worse cognitive functioning, but this worse functioning would be more pronounced for Mexican American individuals than non-Hispanic White individuals.

## Methods

### Participants

Data for this cross-sectional study were obtained from the Health and Aging Brain Study–Health Disparities (HABS-HD) study (formerly the Health and Aging Brain Among Latino Elders [HABLE] study), a longitudinal community-based study examining health disparities in mild cognitive impairment and dementia among Mexican American individuals compared with non-Hispanic White individuals.^29,30,31^ The HABS-HD study methods have been published elsewhere.^30^ The HABS-HD study is a single-site project, and participants were recruited from the Dallas/Fort Worth metropolitan area of Texas using community-based participatory research methods. The HABS-HD study was approved by the institutional review board of the University of North Texas Health Science Center. Each participant (or their legal representative) provided written informed consent, including consent to use and publish their data in future studies. The current study used baseline data from Mexican American and non-Hispanic White individuals 50 years and older who participated in the HABS-HD study from September 1, 2017, through May 31, 2022. This study followed the Strengthening the Reporting of Observational Studies in Epidemiology (STROBE) reporting guideline for cross-sectional studies.

### Procedures

All aspects of the HABS-HD study protocol were conducted in Spanish or English according to participant preference. An interview was conducted, during which data on self-reported race and ethnicity, educational level, sex, annual household income, and current residence were collected. An informant interview by clinicians with expertise in dementia was also conducted using the Clinical Dementia Rating scale^32^ to evaluate functional decline. Data from the Clinical Dementia Rating scale, self- and informant-reported daily function, and neuropsychological testing results were reviewed via consensus conference (including J.R.H., M.P., and S.E.O.) to determine 1 of 3 clinical diagnoses: normal control, mild cognitive impairment, or dementia. As of June 6, 2022, a total of 1614 participants (853 Mexican American and 761 non-Hispanic White individuals) underwent consensus review and had requisite data available for geocoding and ADI linkage.

#### Neuropsychological Assessment

Participants completed a battery of neuropsychological tests. Raw scores from the primary indices of cognitive measures were used in analyses, resulting in 11 total outcomes. Cognitive measures included the Wechsler Memory Scale, third edition (WMS-III) Digit Span Forward subscale (range, 0-16, with higher scores indicating better attention performance), WMS-III Digit Span Backward subscale (range, 0-14, with higher scores indicating better working memory performance), WMS-III Logical Memory 1 subscale (range, 0-75, with higher scores indicating better immediate story recall performance),^33^ WMS-III Logical Memory 2 subscale (range, 0-50, with higher scores indicating better delayed story recall performance), Digit Symbol Substitution Test (DSST; range, 0-93, with higher scores indicating better processing speed performance),^34^ Trail Making Test (TMT) parts A and B (scored by length of time [up to 180 seconds for part A and 300 seconds for part B] taken to complete the test, with longer times indicating worse processing speed [part A] or worse executive functioning [part B] performance),^35^ Spanish-English Verbal Learning Test (SEVLT) Learning subscale (range, 0-60, with higher scores indicating better word list learning performance), SEVLT Delayed Recall subscale (range, 0-15, with higher scores indicating better delayed word list learning performance),^36^ Animal Naming (scored by number of animal names verbalized within 60 seconds, with higher numbers indicating better semantic fluency performance),^34^ and Letter Fluency (FAS; scored by number of words beginning with F, A, and S verbalized within 60 seconds, with higher numbers indicating better phonemic fluency performance).^37^ Histograms of the cognitive data and frequency of missing data for each variable are shown in eFigures 1 and 2 in Supplement 1.

#### Area Deprivation Index

Neighborhood disadvantage was measured by the ADI, which has been made freely available to the public by the University of Wisconsin via the Neighborhood Atlas.^10,17,38^ Each participant’s current residence was geocoded and linked to 2019 ADI scores. For this study, Texas state decile ranks for ADIs were used, which were collapsed into quintiles to reduce the number of comparisons required, with quintile 1 representing the least disadvantaged areas and quintile 5 representing the most disadvantaged areas.

### Statistical Analysis

Statistical analyses were conducted using IBM SPSS Statistics software, version 23 (IBM Corporation). Associations between ADI and cognition were evaluated using separate linear regression models for each cognitive variable, adjusting for group differences in demographic characteristics known to alter cognition, including age, sex, and educational level. The linear regression models were stratified by ethnic group (ie, run separately for older Mexican American and older non-Hispanic White adults). The ADI quintiles were dummy coded, using ADI quintile 1 as the referent, to allow for representation of each quintile group within the regression models. Inverse probability weights (IPWs) were estimated using the ipw package, version 1.2, in R software, version 4.3.0 (R Foundation for Statistical Computing), to address missing cognitive data, then included as weights in the linear regression models. Tests were 2-tailed, and statistical significance was set at 2-tailed *P* < .05 for all analyses, without adjustment for multiple comparisons given the preliminary nature of this investigation. Results were expressed as means with SDs and regression coefficients (β estimates) with corresponding 95% CIs.

## Results

From September 2017 through May 2022, 1614 individuals (mean [SD] age, 66.3 [8.7] years; 980 women [60.7%] and 634 men [39.3%]) participated in the HABS-HD study and had available ADI data. Of those, 853 participants (52.9%) were Mexican American (mean [SD] age, 63.9 [7.9] years; 566 women [66.4%] and 287 men [33.6%]), and 761 (47.1%) were non-Hispanic White (mean [SD] age, 69.1 [8.7] years; 414 women [54.4%] and 347 men [45.6%]). Among Mexican American participants, 71 (8.3%) lived in ADI quintile 1 (least disadvantaged area), 106 (12.4%) in quintile 2, 132 (15.5%) in quintile 3, 264 (30.9%) in quintile 4, and 280 (32.8%) in quintile 5 (most disadvantaged area). Among non-Hispanic White participants, 296 (38.9%) lived in ADI quintile 1, 235 (30.9%) in quintile 2, 119 (15.6%) in quintile 3, 85 (11.2%) in quintile 4, and 26 (3.4%) in quintile 5. Overall, 80 Mexican American participants (9.4%) had a dementia diagnosis (4 individuals in ADI quintile 1, 5 in quintile 2, 5 in quintile 3, 40 in quintile 4, and 26 in quintile 5) compared with 46 non-Hispanic White participants (6.0%; 16 individuals in ADI quintile 1, 16 in quintile 2, 8 in quintile 3, 6 in quintile 4, and 0 in quintile 5). Additional demographic and clinical characteristics for both ethnic groups by ADI quintile are shown in Table 1.

**Table 1.** Participant Demographic and Clinical Characteristics by ADI Quintile and Ethnicity

Characteristic	Participants, No. (%)				
	ADI 1	ADI 2	ADI 3	ADI 4	ADI 5
**Mexican American adults (n = 853)**					
Total participants, No.	71	106	132	264	280
Age, y					
50-59	21 (29.6)	25 (23.6)	39 (29.5)	93 (35.2)	88 (31.4)
60-69	30 (42.3)	62 (58.5)	58 (43.9)	107 (40.5)	123 (43.9)
70-79	17 (23.9)	16 (15.1)	32 (24.2)	56 (21.2)	59 (21.1)
≥80	3 (4.2)	3 (2.8)	3 (2.3)	8 (3.0)	10 (3.6)
Educational level					
Grade 8 or lower	11 (15.5)	20 (18.9)	45 (34.1)	115 (43.6)	161 (57.5)
Grades 9-12	19 (26.8)	31 (29.2)	44 (33.3)	99 (37.5)	89 (31.8)
Some college	17 (23.9)	21 (19.8)	28 (21.2)	31 (11.7)	19 (6.8)
College graduate or higher	24 (33.8)	34 (32.1)	15 (11.4)	19 (7.2)	11 (3.9)
Household income, $					
0-24 999	21 (29.6)	27 (25.5)	55 (41.7)	141 (53.4)	180 (64.3)
25 000-49 999	13 (18.3)	24 (22.6)	39 (29.5)	84 (31.8)	70 (25.0)
50 000-74 999	9 (12.7)	20 (18.9)	20 (15.2)	17 (6.4)	14 (5.0)
75 000-99 999	4 (5.6)	16 (15.1)	11 (8.3)	7 (2.7)	5 (1.8)
≥100 000	23 (32.4)	19 (17.9)	5 (3.8)	4 (1.5)	1 (0.4)
Missing	1 (1.4)	0	2 (1.5)	11 (4.2)	10 (3.6)
Sex					
Female	50 (70.4)	60 (56.6)	88 (66.7)	180 (68.2)	188 (67.1)
Male	21 (29.6)	46 (43.4)	44 (33.3)	84 (31.8)	92 (32.9)
Diagnosis					
Cognitively normal	57 (80.3)	87 (82.1)	102 (77.3)	190 (72.0)	199 (71.1)
Mild cognitive impairment	10 (14.1)	14 (13.2)	25 (18.9)	54 (20.5)	55 (19.6)
Dementia	4 (5.6)	5 (4.7)	5 (3.8)	40 (15.2)	26 (9.3)
**Non-Hispanic White adults (n = 761)**					
Total participants, No.	296	235	119	85	26
Age, y					
50-59	44 (14.9)	32 (13.6)	19 (16.0)	17 (20.0)	5 (19.2)
60-69	84 (28.4)	79 (33.6)	43 (36.1)	34 (40.0)	10 (38.5)
70-79	143 (48.3)	88 (37.4)	37 (31.1)	20 (23.5)	10 (38.5)
≥80	25 (8.4)	36 (15.3)	20 (16.8)	14 (16.5)	1 (3.8)
Educational level					
Grade 8 or lower	0	1 (0.4)	1 (0.8)	1 (1.2)	2 (7.7)
Grades 9-12	21 (7.1)	28 (11.9)	21 (17.6)	23 (27.1)	9 (34.6)
Some college	67 (22.6)	61 (26.0)	49 (41.2)	31 (36.5)	7 (26.9)
College graduate or higher	208 (70.3)	145 (61.7)	48 (40.3)	30 (35.3)	8 (30.8)
Household income, $					
0-24 999	17 (5.7)	19 (8.1)	20 (16.8)	35 (41.2)	12 (46.2)
25 000-49 999	43 (14.5)	48 (20.4)	26 (21.8)	26 (30.6)	7 (26.9)
50 000-74 999	63 (21.3)	60 (25.5)	23 (19.3)	13 (15.3)	5 (19.2)
75 000-99 999	42 (14.2)	40 (17.0)	24 (20.2)	2 (2.4)	0
≥100 000	124 (41.9)	63 (26.8)	23 (19.3)	7 (8.2)	2 (7.7)
Missing	7 (2.4)	5 (2.1)	3 (2.5)	2 (2.4)	0
Sex					
Female	154 (52.0)	128 (54.5)	63 (52.9)	56 (65.9)	13 (50.0)
Male	142 (48.0)	107 (45.5)	56 (47.1)	29 (34.1)	13 (50.0)
Diagnosis					
Cognitively normal	255 (86.1)	194 (82.6)	91 (76.5)	64 (75.3)	21 (80.8)
Mild cognitive impairment	25 (8.4)	25 (10.6)	20 (16.8)	15 (17.6)	5 (19.2)
Dementia	16 (5.4)	16 (6.8)	8 (6.7)	6 (7.1)	0

Abbreviation: ADI, Area Deprivation Index quintile.

In demographically adjusted linear regression models, older Mexican American adults residing in ADI quintile 1 had better performance than those residing in more disadvantaged ADI quintiles (ie, quintiles 3-5) on 7 of 11 cognitive measures, including SEVLT Learning (ADI quintile 5: β = −2.50 [95% CI, −4.46 to –0.54]), SEVLT Delayed Recall (ADI quintile 3: β = −1.11 [95% CI, −1.97 to –0.24]; ADI quintile 4: β = −1.05 [95% CI, −1.85 to –0.25]; ADI quintile 5: β = −0.97 [95% CI, −1.78 to –0.16]), WMS-III Digit Span Forward (ADI quintile 3: β = −1.09 [95% CI, −1.60 to –0.59]; ADI quintile 4: β = −1.14 [95% CI, −1.60 to –0.67]; ADI quintile 5: β = −1.07 [95% CI, −1.54 to –0.59]), TMT part A (ADI quintile 5: β = 7.85; 95% CI, 1.28-14.42), TMT part B (ADI quintile 4: β = 26.17 [95% CI, 6.85-45.48]; ADI quintile 5: β = 31.75 [95% CI, 12.16-51.35]), FAS (ADI quintile 4: β = −0.12; 95% CI, −5.39 to −0.43), and DSST (ADI quintile 3: β = −3.73 [95% CI, −6.20 to –1.26]; ADI quintile 4: β = −4.44 [95% CI, −6.72 to –2.16]; ADI quintile 5: β = −4.45 [95% CI, −6.77 to –2.14]) (Table 2). No significant differences in cognition were found between older Mexican American adults living in ADI quintile 2 vs quintile 1.

**Table 2.** Linear Regression Analysis for Older Mexican American Adults^a^

Outcome measure	Score, mean (SD)	β (95% CI)	*F* (*df*)	*P* value
SEVLT Learning^b^	NA	NA	39.79 (7 to 843)	<.001
ADI 1	32.32 (9.43)	NA	NA	NA
ADI 2	31.32 (8.38)	0.12 (−2.05 to 2.28)	NA	.92
ADI 3	28.11 (7.40)	−1.87 (−3.96 to 0.21)	NA	.08
ADI 4	24.72 (7.24)	−1.83 (−3.76 to 0.10)	NA	.06
ADI 5	26.68 (9.05)	−2.50 (−4.46 to −0.54)	NA	.01
SEVLT Delayed Recall^b^	NA	NA	35.86 (7 to 816)	<.001
ADI 1	8.49 (3.44)	NA	NA	NA
ADI 2	7.80 (3.12)	−0.24 (−1.14 to 0.65)	NA	.59
ADI 3	6.52 (3.09)	−0.11 (−1.97 to −0.24)	NA	.01
ADI 4	5.32 (3.08)	−1.05 (−1.85 to −0.25)	NA	.01
ADI 5	5.94 (3.89)	−0.97 (−1.78 to −0.16)	NA	.02
WMS-III Logical Memory 1^c^	NA	NA	30.12 (7 to 841)	<.001
ADI 1	33.65 (11.41)	NA	NA	NA
ADI 2	32.54 (10.12)	0.89 (−1.95 to 3.72)	NA	.54
ADI 3	30.11 (10.75)	0.10 (−2.79 to 2.83)	NA	.94
ADI 4	26.96 (10.30)	0.15 (−2.39 to 2.68)	NA	.91
ADI 5	28.08 (10.76)	−0.19 (−2.76 to 2.37)	NA	.88
WMS-III Logical Memory 2^c^	NA	NA	29.73 (7 to 841)	<.001
ADI 1	20.59 (8.49)	NA	NA	NA
ADI 2	19.21 (7.68)	0.40 (−1.78 to 2.59)	NA	.72
ADI 3	18.15 (8.21)	0.11 (−2.00 to 2.22)	NA	.92
ADI 4	15.43 (7.72)	0.23 (−1.72 to 2.18)	NA	.82
ADI 5	16.56 (8.01)	−0.13 (−2.11 to 1.84)	NA	.90
WMS-III Digit Span Forward^d^	NA	NA	43.69 (7 to 836)	<.001
ADI 1	8.48 (2.35)	NA	NA	NA
ADI 2	7.63 (2.25)	−0.50 (−1.02 to 0.02)	NA	.06
ADI 3	6.94 (2.01)	−1.09 (−1.60 to −0.59)	NA	<.001
ADI 4	6.39 (1.56)	−1.14 (−1.60 to −0.67)	NA	<.001
ADI 5	6.44 (1.79)	−1.07 (−1.54 to −0.59)	NA	<.001
WMS-III Digit Span Backward^d^	NA	NA	32.81 (7 to 838)	<.001
ADI 1	5.35 (2.24)	NA	NA	NA
ADI 2	4.97 (1.90)	−0.11 (−0.64 to 0.42)	NA	.68
ADI 3	4.71 (2.01)	−0.30 (−0.81 to 0.21)	NA	.25
ADI 4	3.88 (1.87)	−0.44 (−0.91 to 0.04)	NA	.07
ADI 5	4.00 (1.69)	−0.48 (−0.97 to −0.003)	NA	.05
TMT part A, s^e^	NA	NA	61.53 (7 to 838)	<.001
ADI 1	36.57 (19.44)	NA	NA	NA
ADI 2	44.14 (25.00)	3.32 (−3.92 to 10.56)	NA	.90
ADI 3	48.42 (26.24)	3.46 (−3.53 to 10.45)	NA	.97
ADI 4	59.75 (31.52)	4.46 (−2.02 to 10.94)	NA	.18
ADI 5	64.48 (37.28)	7.85 (1.28 to 14.42)	NA	.02
TMT part B, s^e^	NA	NA	96.30 (7 to 818)	<.001
ADI 1	103.17 (69.11)	NA	NA	NA
ADI 2	126.89 (81.82)	5.33 (−16.24 to 26.89)	NA	.63
ADI 3	154.45 (89.24)	13.49 (−7.40 to 34.37)	NA	.21
ADI 4	211.37 (88.73)	26.17 (6.85 to 45.48)	NA	.008
ADI 5	199.23 (94.31)	7.85 (12.16 to 51.35)	NA	.002
Letter Fluency total^f^	NA	NA	53.32 (7 to 844)	<.001
ADI 1	32.77 (11.69)	NA	NA	NA
ADI 2	31.10 (12.62)	0.10 (−2.68 to 2.88)	NA	.07
ADI 3	28.42 (11.55)	−1.21 (−3.89 to 1.47)	NA	.38
ADI 4	23.14 (9.21)	−2.91 (−5.39 to −0.43)	NA	.02
ADI 5	25.06 (9.89)	−1.97 (−4.49 to 0.55)	NA	.12
Animal Naming total^g^	NA	NA	37.21 (7 to 845)	<.001
ADI 1	18.06 (4.79)	NA	NA	NA
ADI 2	17.94 (5.44)	0.31 (−0.96 to 1.57)	NA	.64
ADI 3	16.62 (4.96)	−0.24 (−1.46 to 0.98)	NA	.70
ADI 4	14.30 (4.61)	−0.94 (−2.07 to 0.20)	NA	.11
ADI 5	14.92 (4.82)	−1.10 (−2.25 to 0.05)	NA	.06
DSST total^h^	NA	NA	180.52 (7 to 838)	<.001
ADI 1	44.10 (12.31)	NA	NA	NA
ADI 2	40.39 (13.18)	−0.93 (−3.49 to 1.63)	NA	.48
ADI 3	35.34 (14.15)	−3.73 (−6.20 to −1.26)	NA	.003
ADI 4	27.17 (11.38)	−4.44 (−6.72 to −2.16)	NA	<.001
ADI 5	28.90 (11.45)	−4.45 (−6.77 to −2.14)	NA	<.001

Abbreviations: ADI, Area Deprivation Index quintile; DSST, Digit Symbol Substitution Test; NA, not applicable; SEVLT, Spanish-English Verbal Learning Test; TMT, Trail Making Test; WMS-III, Wechsler Memory Scale, third edition.

^a^ All models were adjusted for age, educational level, and sex and weighted by inverse probability weights for each outcome variable to address missing data; ADI quintile 1 was considered the referent.

^b^ Learning: range, 0 to 60, with higher scores indicating better word list learning performance. Delayed Recall: range, 0 to 15, with higher scores indicating better delayed word list learning performance.

^c^ Logical Memory 1: range, 0 to 75, with higher scores indicating better immediate story recall performance. Logical Memory 2: range, 0 to 50, with higher scores indicating better delayed story recall performance.

^d^ Digit Span Forward: range, 0 to 16, with higher scores indicating better attention performance. Digit Span Backward: range, 0 to 14, with higher scores indicating better working memory performance.

^e^ Scored by length of time (up to 180 seconds for part A and 300 seconds for part B) taken to complete the test, with longer times indicating worse processing speed (part A) or worse executive functioning (part B) performance.

^f^ Scored by number of words beginning with F, A, and S verbalized within 60 seconds, with higher numbers indicating better phonemic fluency performance.

^g^ Scored by number of animal names verbalized within 60 seconds, with higher numbers indicating better semantic fluency performance.

^h^ Range, 0 to 93, with higher scores indicating better processing speed performance.

Older non-Hispanic White adults residing in ADI quintile 1 had better performance than their counterparts residing in ADI quintile 4 on 4 of 11 cognitive measures, including SEVLT Learning (β = −2.35; 95% CI, −4.40 to –0.30), SEVLT Delayed Recall (β = −0.95; 95% CI, −1.73 to –0.17), TMT part B (β = 15.95; 95% CI, 2.47-29.44), and DSST (β = −3.96; 95% CI, −6.49 to –1.43) (Table 3). No significant differences in cognitive performance were found between older non-Hispanic White adults living in ADI quintiles 2, 3, or 5 vs quintile 1.

**Table 3.** Linear Regression Analysis for Older Non-Hispanic White Adults^a^

Outcome measure	Score, mean (SD)	β (95% CI)	*F* (*df*)	*P* value
SEVLT Learning^b^	NA	NA	34.70 (7 to 752)	<.001
ADI 1	33.56 (9.46)	NA	NA	NA
ADI 2	31.79 (9.96)	−0.79 (−2.22 to 0.63)	NA	.28
ADI 3	31.40 (9.31)	−0.90 (−2.68 to 0.90)	NA	.33
ADI 4	31.62 (9.47)	−2.35 (−4.40 to −0.30)	NA	.03
ADI 5	31.26 (9.54)	−1.72 (−5.10 to 1.67)	NA	.32
SEVLT Delayed Recall^b^	NA	NA	27.73 (7 to 741)	<.001
ADI 1	8.63 (3.34)	NA	NA	NA
ADI 2	8.06 (3.57)	−0.30 (−0.85 to 0.24)	NA	.27
ADI 3	7.08 (3.57)	−0.65 (−1.32 to 0.03)	NA	.06
ADI 4	7.80 (3.69)	−0.95 (−1.73 to −0.17)	NA	.02
ADI 5	7.70 (3.59)	−0.46 (−1.74 to 0.82)	NA	.48
WMS-III Logical Memory 1^c^	NA	NA	16.35 (7 to 753)	<.001
ADI 1	41.23 (11.63	NA	NA	NA
ADI 2	39.12 (12.57)	−0.76 (−2.63 to 1.10)	NA	.42
ADI 3	38.92 (12.13)	−0.45 (−2.79 to 1.90)	NA	.71
ADI 4	38.68 (11.72)	−2.35 (−5.03 to 0.34)	NA	.09
ADI 5	37.96 (11.35)	−3.70 (−8.12 to 0.73)	NA	.10
WMS-III Logical Memory 2^c^	NA	NA	17.20 (7 to 753)	<.001
ADI 1	25.16 (8.73)	NA	NA	NA
ADI 2	23.55 (9.47)	−0.73 (−2.15 to 0.70)	NA	.32
ADI 3	23.30 (9.20)	−0.41 (−2.20 to 1.39)	NA	.66
ADI 4	23.32 (9.39)	−1.70 (−3.75 to 0.36)	NA	.11
ADI 5	22.83 (8.73)	−2.66 (−6.04 to 0.73)	NA	.12
WMS-III Digit Span Forward^d^	NA	NA	7.25 (7 to 750)	<.001
ADI 1	9.86 (2.05)	NA	NA	NA
ADI 2	9.57 (2.16)	−0.13 (−0.48 to 0.22)	NA	.46
ADI 3	9.53 (2.21)	−0.02 (−0.46 to 0.42)	NA	.92
ADI 4	9.39 (2.22)	−0.49 (−0.99 to 0.01)	NA	.06
ADI 5	9.36 (2.20)	0.15 (−0.67 to 0.96)	NA	.73
WMS-III Digit Span Backward^d^	NA	NA	6.28 (7 to 748)	<.001
ADI 1	6.52 (2.02)	NA	NA	NA
ADI 2	6.33 (2.25)	−0.02 (−0.37 to 0.33)	NA	.91
ADI 3	6.27 (2.13)	−0.08 (−0.52 to 0.36)	NA	.72
ADI 4	6.16 (2.08)	−0.03 (−0.80 to 0.20)	NA	.23
ADI 5	6.09 (2.03)	0.001 (−0.83 to 0.83)	NA	>.99
TMT part A, s^e^	NA	NA	15.94 (7 to 749)	<.001
ADI 1	35.40 (18.30	NA	NA	NA
ADI 2	37.95 (19.65	1.27 (−1.60 to 4.14)	NA	.39
ADI 3	38.21 (19.10	0.16 (−3.46 to 3.77)	NA	.93
ADI 4	36.90 (17.13	1.33 (−2.81 to 5.47)	NA	.53
ADI 5	37.54 (17.03	−0.37 (−7.19 to 6.45)	NA	.91
TMT part B, s^e^	NA	NA	25.54 (7 to 745)	<.001
ADI 1	88.48 (55.72)	NA	NA	NA
ADI 2	95.85 (59.39)	−0.40 (−9.75 to 8.95)	NA	.93
ADI 3	100.33 (62.09)	8.24 (−3.56 to 20.04)	NA	.17
ADI 4	102.04 (65.24)	15.95 (2.47 to 29.44)	NA	.02
ADI 5	104.93 (66.24)	8.79 (−13.41 to 30.99)	NA	.44
Letter Fluency total^f^	NA	NA	14.89 (7 to 752)	<.001
ADI 1	37.98 (11.32)	NA	NA	NA
ADI 2	37.61 (12.15)	0.78 (−1.08 to 2.64)	NA	.41
ADI 3	36.09 (11.90)	−1.34 (−3.68 to 1.00)	NA	.26
ADI 4	35.24 (11.30)	−1.95 (−4.63 to 0.73)	NA	.15
ADI 5	35.16 (11.54)	0.44 (−3.98 to 4.85)	NA	.85
Animal Naming^g^	NA	NA	17.38 (7 to 752)	<.001
ADI 1	18.91 (5.21)	NA	NA	NA
ADI 2	18.51 (5.50)	0.38 (−0.46 to 1.22)	NA	.37
ADI 3	18.38 (5.27)	−0.06 (−1.11 to 0.99)	NA	.91
ADI 4	18.39 (5.22)	−0.12 (−1.32 to 1.09)	NA	.85
ADI 5	18.14 (5.23)	0.21 (−1.77 to 2.20)	NA	.83
DSST total^h^	NA	NA	34.46 (7 to 748)	<.001
ADI 1	46.50 (11.40)	NA	NA	NA
ADI 2	44.93 (12.28)	0.27 (−1.49 to 2.02)	NA	.77
ADI 3	43.88 (11.25)	−1.43 (−3.65 to 0.78)	NA	.20
ADI 4	43.76 (10.88)	−3.96 (−6.49 to −1.43)	NA	.002
ADI 5	42.94 (11.10)	−1.51 (−5.67 to 2.65)	NA	.48

Abbreviations: ADI, Area Deprivation Index quintile; DSST, Digit Symbol Substitution Test; NA, not applicable; SEVLT, Spanish-English Verbal Learning Test; TMT, Trail Making Test; WMS-III, Wechsler Memory Scale, third edition.

^a^ All models were adjusted for age, educational level, and sex and weighted by inverse probability weights for each outcome variable to address missing data; ADI quintile 1 was considered the referent.

^b^ SEVLT Learning: range, 0 to 60, with higher scores indicating better word list learning performance. Delayed Recall: range, 0 to 15, with higher scores indicating better delayed word list learning performance.

^c^ Logical Memory 1: range, 0 to 75, with higher scores indicating better immediate story recall performance. Logical Memory 2: range, 0 to 50, with higher scores indicating better delayed story recall performance.

^d^ Digit Span Forward: range, 0 to 16, with higher scores indicating better attention performance. Digit Span Backward: range, 0 to 14, with higher scores indicating better working memory performance.

^e^ Scored by length of time (up to 180 seconds for part A and 300 seconds for part B) taken to complete the test, with longer times indicating worse processing speed (part A) or worse executive functioning (part B) performance.

^f^ Scored by number of words beginning with F, A, and S verbalized within 60 seconds, with higher numbers indicating better phonemic fluency performance.

^g^ Scored by number of animal names verbalized within 60 seconds, with higher numbers indicating better semantic fluency performance.

^h^ Range, 0 to 93, with higher scores indicating better processing speed performance.

## Discussion

To our knowledge, this cross-sectional study reflects one of the largest examinations of neighborhood context and neuropsychological functioning among a community-based multiethnic cohort of older adults. Our findings revealed an association between higher neighborhood-level disadvantage (ADI quintiles 3-5) and worse performance on tests of memory, attention, processing speed, and executive functioning among older Mexican American adults after controlling for individual-level demographic factors. In contrast, only older non-Hispanic White adults living in ADI quintile 4 had worse performance than their counterparts living in ADI quintile 1 on 4 measures (SEVLT Learning, SELVT Delayed Recall, TMT part B, and DSST). These findings were consistent with previous research^24,25^ revealing the association between neighborhood context and cognition may vary by racial and ethnic background. However, another study^19^ found that the association between ND and significant cognitive decline remained after accounting for race.

Differences between levels of ND in these areas of cognition (and in some cases, the same tests) have been found in previous studies,^19,25^ suggesting that these cognitive areas may be particularly sensitive to the consequences of contextual-level social determinants of health. For example, in the current study, ND was associated with the DSST, a measure of processing speed, in both ethnic groups, and several measures associated with ADI quintile in the Mexican American group involved a speed component (eg, FAS and TMT parts A and B). Processing speed can be altered by different conditions, including vascular disease, which may be disproportionately impacted by ND in racial and ethnic minority groups.^15^ Nevertheless, ADI was not associated with all measures of processing speed, and additional research examining which cognitive domains are most often associated with ND is needed.

Reasons for the differential association of ND with cognition among older Mexican American and non-Hispanic White adults are not fully understood. There is likely an interplay of individual, sociopolitical, and environmental factors, which may have a role in how ND is associated with cognition among different racial and ethnic groups. For example, the potential negative implications of ND for health outcomes may be compounded by perceived discrimination and housing discrimination experienced by individuals from marginalized groups. Furthermore, non-Hispanic White adults living in disadvantaged areas may have more opportunities for upward mobility due to fewer barriers related to educational level and employment, which could result in moving out of disadvantaged areas at higher rates compared with individuals from ethnically and racially diverse backgrounds. The linear regression models examining ADI and cognition were adjusted for individual-level demographic factors known to be associated with cognition (ie, age, sex, and educational level),^39,40,41^ but there may be other factors relevant to cognition that were not included in the analyses. Thus, although ADI may capture distinct contextual-level information that can have implications for cognition, there is still heterogeneity within similarly disadvantaged neighborhoods by ethnic group, likely reflective of US structural inequities. Research examining exposure to ND over the life course among different racial and ethnic groups is needed.

There are many potential mechanistic pathways for ND to impact cognitive aging across the life span. Lawrence et al^42^ examined epigenetic aging among 2630 women living in the US and Puerto Rico and reported that those with the highest ND (>75th percentile) vs the lowest ND (≤25th percentile) had higher epigenetic age acceleration as estimated by 3 of 4 epigenetic clocks. The HABS-HD study currently captures broad-based multilevel omics (ie, the structure and function of the whole makeup of a biological function at different levels) and imaging data; therefore, additional work is ongoing to more deeply explore biological and sociocultural mechanistic pathways for the association between neighborhood-level disadvantage and brain aging.

Notably, when compared with those living in the most advantaged neighborhoods (ie, ADI quintile 1), there were only significant differences in cognitive performance for individuals living in areas below the ADI quintile 2; both Mexican American and non-Hispanic White participants living in quintile 2 had cognitive performance similar to those living in the quintile 1. For older Mexican American adults, we found a broader range of ADI quintiles with worse cognitive performance compared with the most advantaged areas than previous research,^19^ which only found baseline differences in ADI quintiles 4 and 5 compared with ADI quintile 1. Although more research is needed to identify the specific communities at risk of adverse cognitive outcomes, evaluating and addressing the factors associated with health disparities in these less advantaged areas (ie, ADI quintiles 3-5) may be beneficial, especially among racial and ethnic minority individuals. These findings also support the notion that context-related risk of cognitive decline does not occur only among those living in the most disadvantaged areas; neighborhoods that have a moderate disadvantage are also in need of resources to address health risks. This issue is particularly important because ND is a fundamentally modifiable factor that is impacted through social policy and community intervention.

### Limitations

This study has limitations. First, the current analyses are cross-sectional. However, the HABS-HD study is longitudinal, and follow-up analyses over time will be conducted after visit 2 assessments have been completed. Second, the number of non-Hispanic White participants residing in the most disadvantaged neighborhoods was small (26 participants in ADI quintile 5), and there were no non-Hispanic White individuals classified as having dementia in ADI quintile 5, which may have had implications for results revealing a lack of a dose-response association between ADI quintile and cognition in the non-Hispanic White group (ie, there were significant differences between ADI quintiles 1 and 4 but not between ADI quintiles 1 and 5). Third, as with most studies examining cognitive aging, there were missing outcome data. Although adjustments were made to address missing data using IPW in analyses, the assumption that data were missing at random may have been violated because some missing data may be associated with more impaired participants not being able to complete the full neuropsychological battery. Fourth, because this cohort was from Texas, socioeconomic disadvantage at the state level (ie, Texas state ADI deciles) was used, which may limit generalizability to populations outside of Texas. Fifth, although not necessarily a limitation, these findings are specific to older Mexican American vs non-Hispanic White adults and cannot be generalized to other racial and ethnic minority groups. The HABS-HD study is currently enrolling a comparable cohort of older African American adults, which will facilitate similar research across the 3 largest racial and ethnic groups in the US.

## Conclusions

Overall, this cross-sectional study represents an important and novel contribution to the AD literature and allows for investigation of the specific implications of individual- and contextual-level disadvantage factors. The study’s findings revealed that aging in a disadvantaged neighborhood is associated with worse cognitive functioning, particularly for older Mexican American adults. In addition to individual demographic factors, neighborhood-level disadvantage accounted for some differences in neuropsychological performance between Mexican American and non-Hispanic White groups. Because race and ethnicity are sociocultural constructs that serve as proxies for other factors that directly play a role in cognitive health disparities,^43^ continuing to understand both individual and contextual factors will be important for improving aging outcomes among diverse populations.

## Notes

Supplement 1. **eFigure 1.** Histograms and Missing Data for Cognitive Tests for Mexican American Group (n = 853) **eFigure 2.** Histograms and Missing Data for Cognitive Tests for Non-Hispanic White Group (n = 761)

Supplement 2.
Members of the HABS-HD Study Team

Supplement 3.
Data Sharing Statement
