# Supplementary material for: Evaluation of Neighborhood-Level Disadvantage and Cognition in Mexican American and Non-Hispanic White Adults 50 Years and Older in the US
Source: JAMA Netw Open. 2023 Aug 30;6(8):e2325325. doi: 10.1001/jamanetworkopen.2023.25325 (PMC10469291; doi:10.1001/jamanetworkopen.2023.25325)
Supplement: Supplement 1. — eFigure 1. Histograms and Missing Data for Cognitive Tests for Mexican American Group (n = 853) eFigure 2. Histograms and Missing Data for Cognitive Tests for Non-Hispanic White Group (n = 761) [file jamanetwopen-e2325325-s001.pdf]

Supplementary Online Content

Wong CG, Miller JB, Zhang F, et al; HABS-HD Study Team. Evaluation of neighborhood-level disadvantage and cognition in Mexican American and non-Hispanic White adults 50 years and older in the US. *JAMA Netw Open*. 2023;6(7):e2325325. doi:10.1001/jamanetworkopen.2023.25325

eFigure 1. Histograms and Missing Data for Cognitive Tests for Mexican American Group (n = 853)

eFigure 2. Histograms and Missing Data for Cognitive Tests for Non-Hispanic White Group (n = 761)

This supplemental material has been provided by the authors to give readers additional information about their work

© 2023 Wong CG et al. *JAMA Network Open*.

eFigure 1. Histograms and Missing Data for Cognitive Tests for Mexican American Group (n = 853)

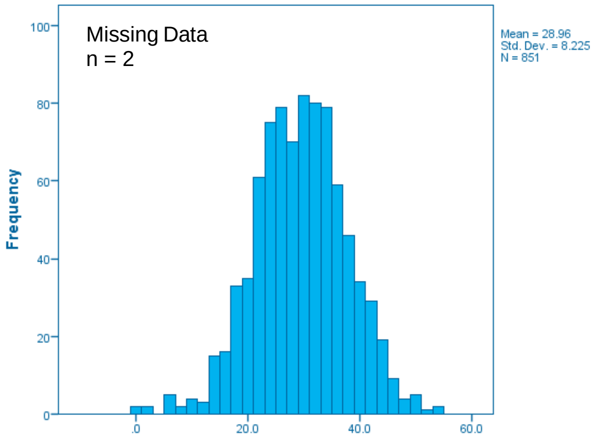

a) SEVLT Learning

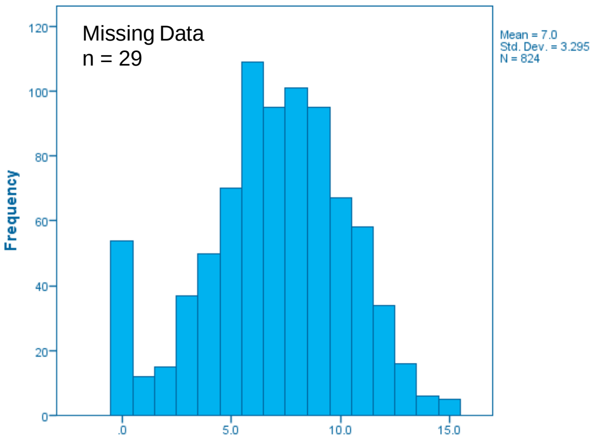

b) SEVLT Delayed Recall

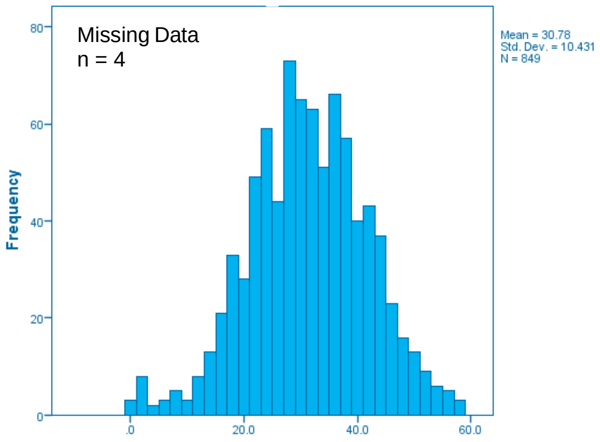

c) Logical Memory I

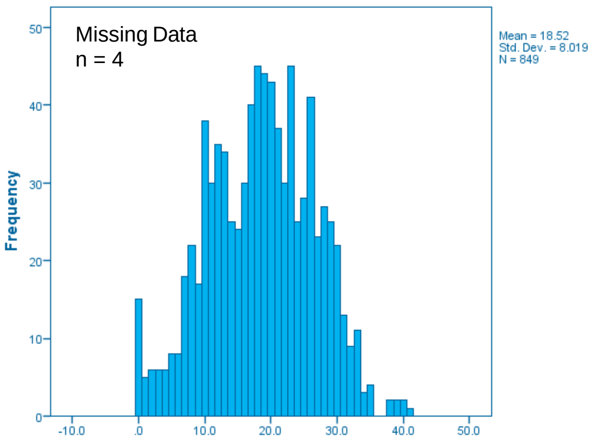

d) Logical Memory II

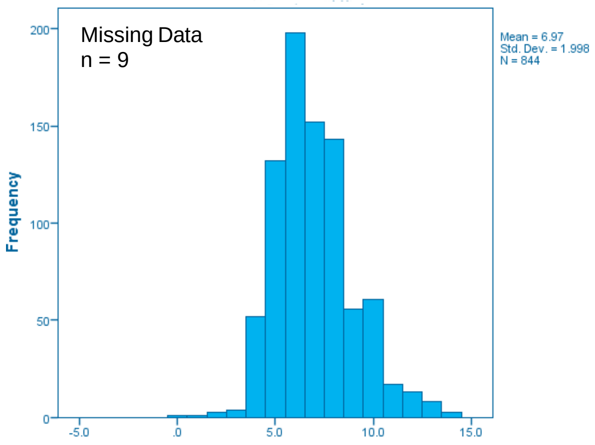

e) Digit Span Forward

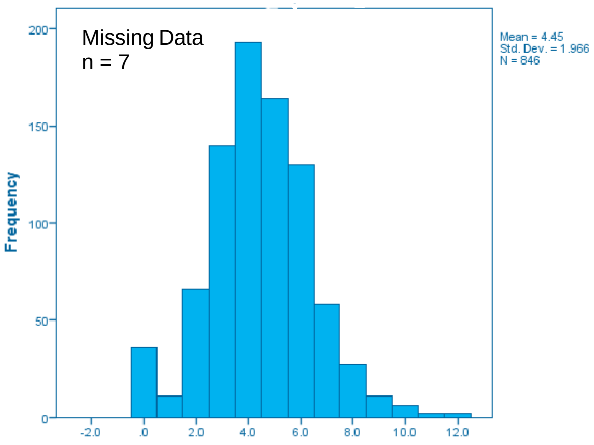

f) Digit Span Backward

© 2023 Wong CG et al. *JAMA Network Open*.

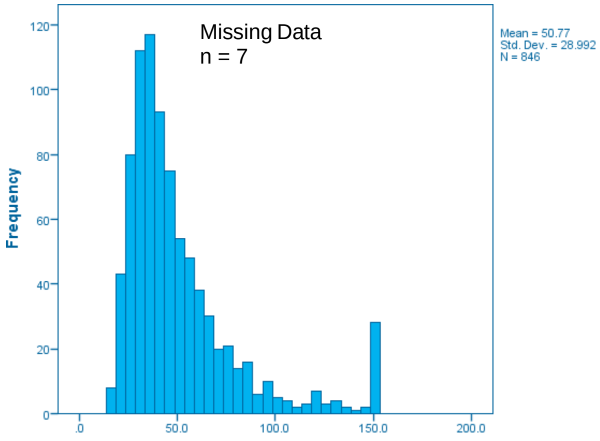

g) Trails A Time

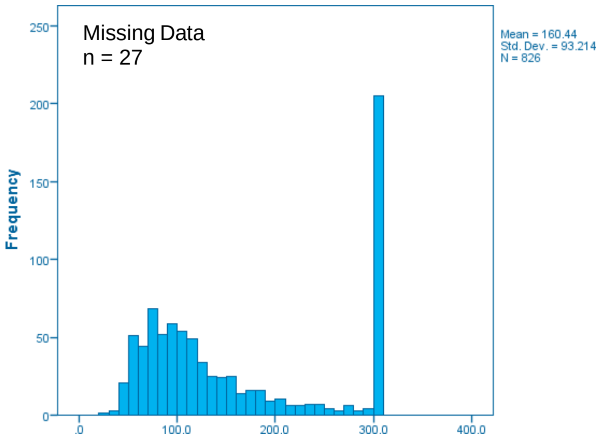

h) Trails B Time

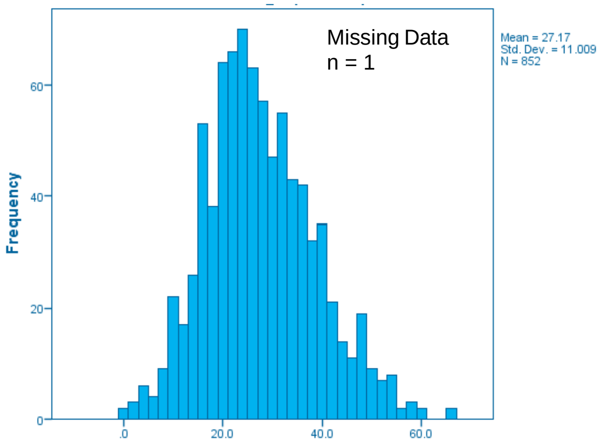

i) FAS Total

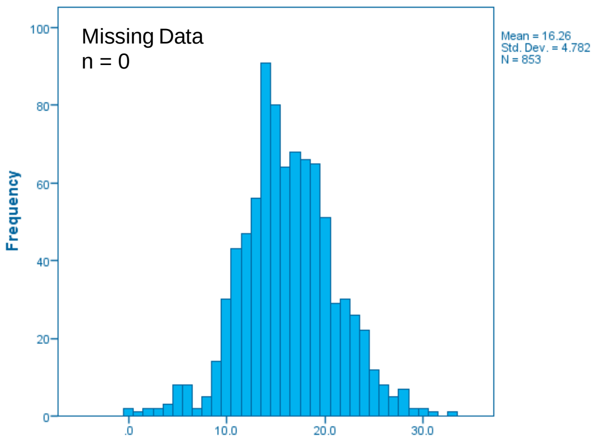

j) Animal Total

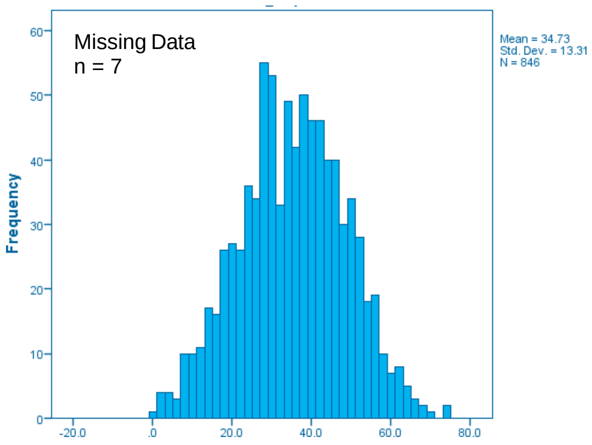

k) Digit Symbol Substitution Test

© 2023 Wong CG et al. *JAMA Network Open*.

eFigure 2. Histograms and Missing Data for Cognitive Tests for Non-Hispanic White Group (n = 761)

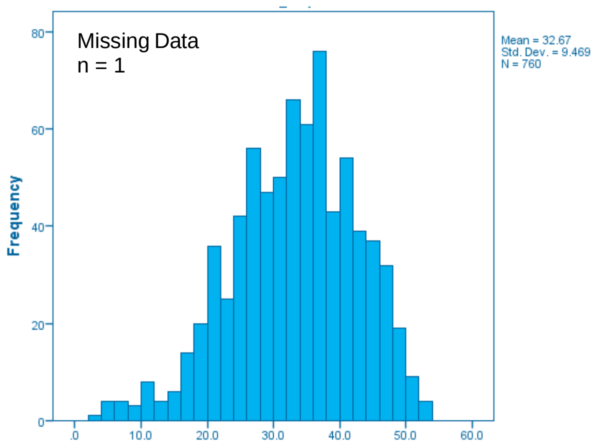

a) SEVLT Learning

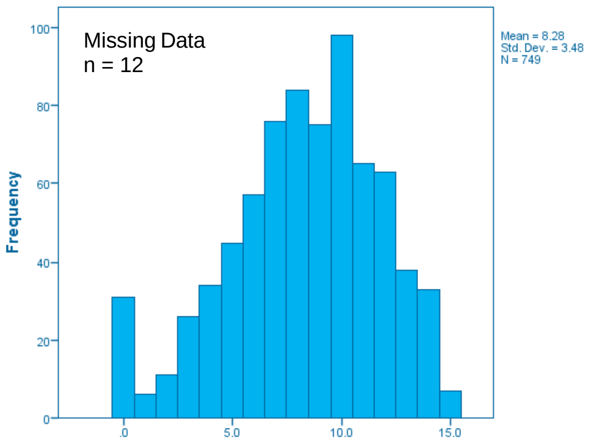

b) SEVLT Delayed Recall

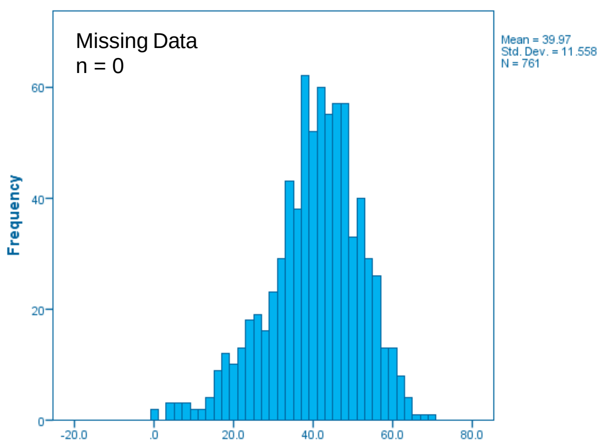

c) Logical Memory I

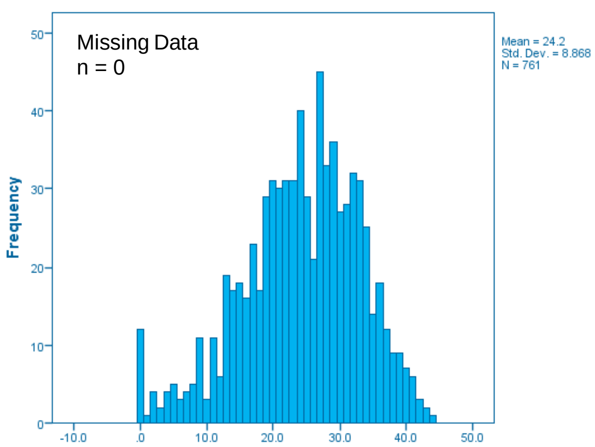

d) Logical Memory II

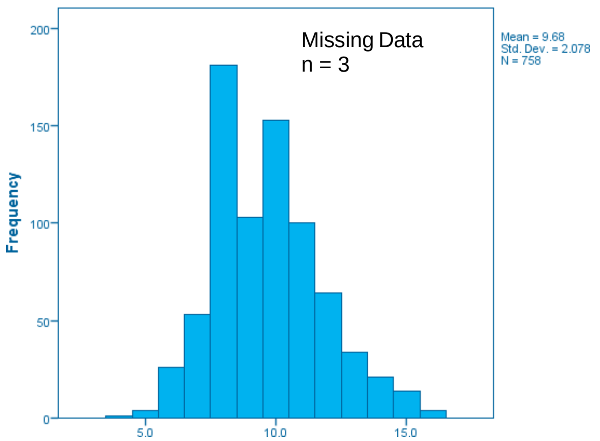

e) Digit Span Forward

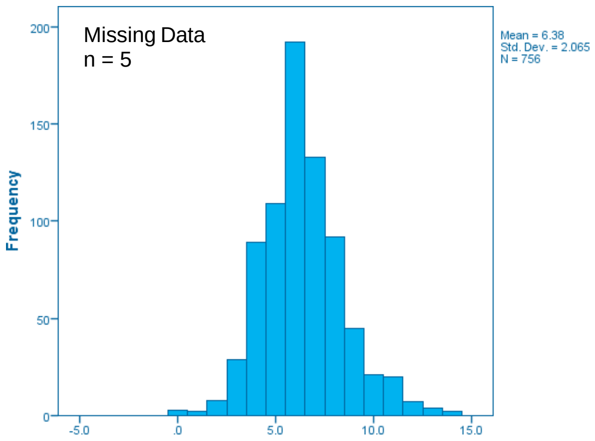

f) Digit Span Backward

© 2023 Wong CG et al. *JAMA Network Open*.

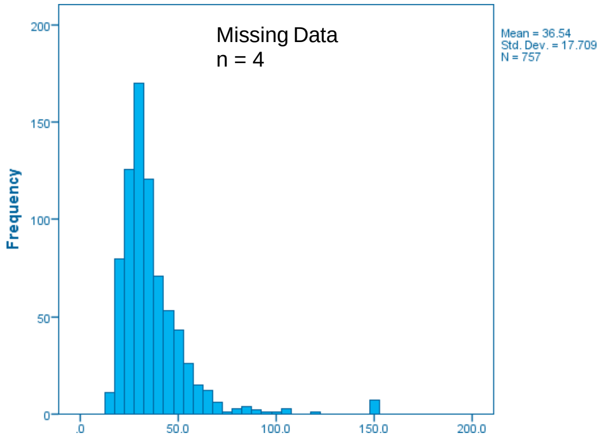

g) Trails A Time

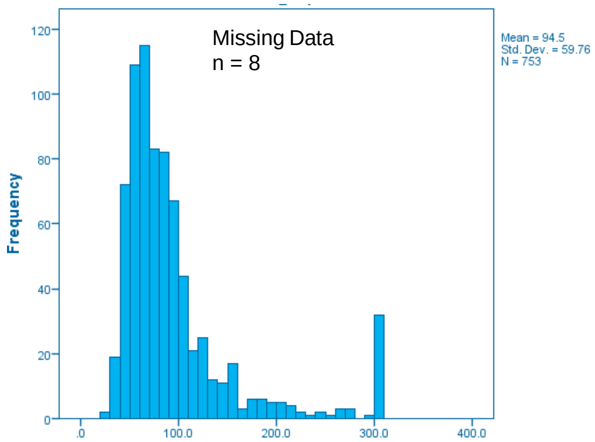

h) Trails B Time

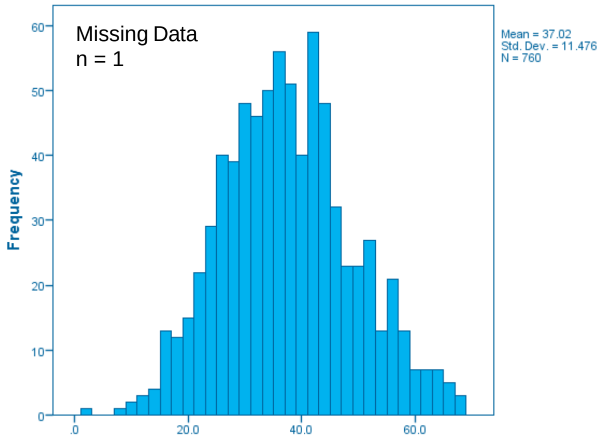

i) FAS Total

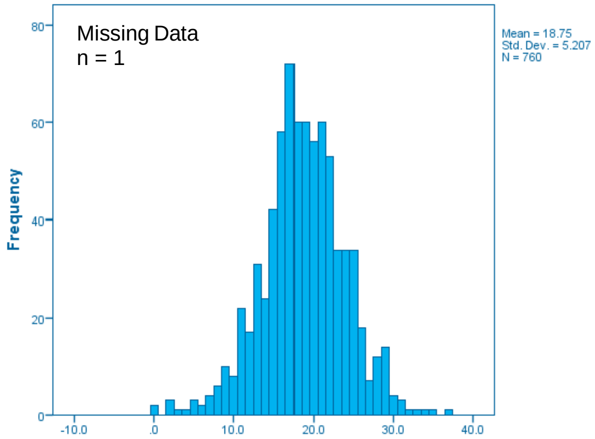

j) Animal Total

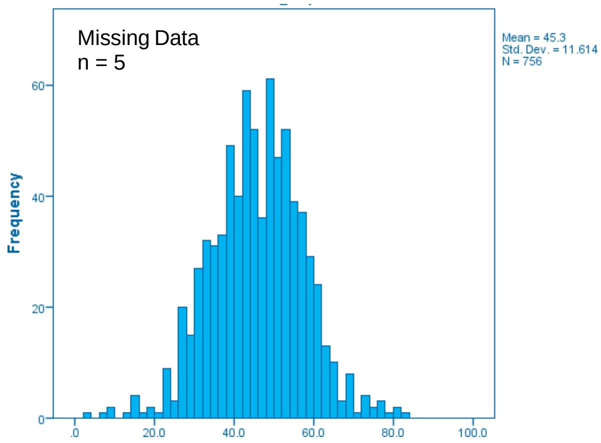

k) Digit Symbol Substitution Test

© 2023 Wong CG et al. *JAMA Network Open*.
