# Supplementary material for: Evaluation of Neighborhood-Level Disadvantage and Cognition in Mexican American and Non-Hispanic White Adults 50 Years and Older in the US
Source: JAMA Netw Open. 2023 Aug 30;6(8):e2325325. doi: 10.1001/jamanetworkopen.2023.25325 (PMC10469291; doi:10.1001/jamanetworkopen.2023.25325)
Supplement: Supplement 2. — Members of the HABS-HD Study Team [file jamanetwopen-e2325325-s002.pdf]

Supplemental Online Content: Nonauthor Collaborators
*First name, last name, and suffix (if applicable) are required and will appear in PubMed.

*Group Name(s): HABS-HD Study Team							
***First Name and Middle Initial(s)**	***Last Name**	***Suffix (eg, Jr, III)**	Academic Degrees	Institution	Location (city, state/province, country)	Role or Contribution, eg, chair, principal investigator	Group (if more than 1 Group listed in the byline) and/or Subgroup (eg, Steering Committee)
Beau	Ances		MD, PhD	Washington University	St. Louis, MO	Co-I	
Ganesh	Babulal		PhD	Washington University	St. Louis, MO	Co-I	
Robert	Barber		PhD	UNT Health Science Center	Fort Worth, TX	Co-I	
Lisa	Barnes		PhD	Rush University Medical Center	Chicago, IL	Co-I	
Matthew	Borzage		PhD	University of Southern California	Los Angeles, CA	Co-I	
Meredith	Braskie		PhD	University of Southern California	Los Angeles, CA	Co-I	
Amrita	Cheema		PhD	Georgetown University Medical Cent	Washington, D.C.	Co-I	
Brad	Christian		PhD	University of Wisconsin - Madison	Madison, WI	Co-I	
Ann	Cohen		PhD	University of Pittsburgh	Pittsburgh, PA	Co-I	
Michael	Donohue		PhD	University of Southern California	Los Angeles, CA	Co-I	
James	Hall		PhD	UNT Health Science Center	Fort Worth, TX	Co-I	
Leigh	Johnson		PhD	UNT Health Science Center	Fort Worth, TX	MPI	
Amy	Kind		PhD	University of Wisconsin - Madison	Madison, WI	Co-I	
Stephanie	Large		PhD	UNT Health Science Center	Fort Worth, TX	Co-I	
Joe	Lee		PhD	Columbia University	New York, NY	Co-I	
Jorge	Llibre-Guerra		MD	Washington University	St. Louis, MO	Co-I	
Mark	Mapstone		PhD	University of California, Irvine	Irvine, CA	Co-I	
David	Mason		DO	UNT Health Science Center	Fort Worth, TX	Co-I	
Michelle	Mielke		PhD	Wake Forest University	Winston-Salem, NC	Co-I	
Rajesh	Nandy		PhD	UNT Health Science Center	Fort Worth, TX	Co-I	
Sid	O'Bryant		PhD	UNT Health Science Center	Fort Worth, TX	Contact PI	
Ozioma	Okonkwo		PhD	University of Wisconsin - Madison	Madison, WI	Co-I	
Ray	Palmer		PhD	The University of Texas Health Scienc	San Antonio, TX	Co-I	
Melissa	Petersen		PhD	UNT Health Science Center	Fort Worth, TX	Co-I	
Nicole	Phillips		PhD	UNT Health Science Center	Fort Worth, TX	Co-I	
Rema	Raman		PhD	University of Southern California	Los Angeles, CA	Co-I	
Robert	Rissman		PhD	University of Southern California	Los Angeles, CA	MPI	
Monica	Rivera Mindt		PhD	Fordham University	New York, NY	Co-I	
Yonggang	Shi		PhD	University of Southern California	Los Angeles, CA	Co-I	

Supplemental Online Content: Nonauthor Collaborators
*First name, last name, and suffix (if applicable) are required and will appear in PubMed.

***First Name and Middle Initial(s)**	***Last Name**	***Suffix (eg, Jr, III)**	Academic Degrees	Institution	Location (city, state/province, country)	Role or Contribution, eg, chair, principal investigator	Group (if more than 1 Group listed in the byline) and/or Subgroup (eg, Steering Committee)
Arthur	Toga		PhD	University of Southern California	Los Angeles, CA	MPI	
Badri	Vardarajan		PhD	Columbia University	New York, NY	Co-I	
Raul	Vintimilla		MD	UNT Health Science Center	Fort Worth, TX	Co-I	
Kristine	Yaffe		MD	University of California, San Francisco	San Francisco, CA	MPI	
Fan	Zhang		PhD	UNT Health Science Center	Fort Worth, TX	Co-I	
Zhengyang	Zhou		PhD	UNT Health Science Center	Fort Worth, TX	Co-I	
