# Supplementary material for: Evaluation of Neighborhood-Level Disadvantage and Cognition in Mexican American and Non-Hispanic White Adults 50 Years and Older in the US
Source: JAMA Netw Open. 2023 Aug 30;6(8):e2325325. doi: 10.1001/jamanetworkopen.2023.25325 (PMC10469291; doi:10.1001/jamanetworkopen.2023.25325)
Supplement: Supplement 3. — Data Sharing Statement [file jamanetwopen-e2325325-s003.pdf]

Data Sharing Statement

Wong. Evaluation of Neighborhood-Level Disadvantage and Cognition in Mexican American and Non-Hispanic White Adults 50 Years and Older in the US. *JAMA Netw Open*. Published July 25, 2023. doi:10.1001/jamanetworkopen.2023.25325

Data
Data available: Yes
Data types: Deidentified participant data, Data dictionary
How to access data: https://apps.unthsc.edu/itr/researchers
When available: beginning date: 06-01-2022

Supporting Documents
Document types: None

Additional Information
Who can access the data: Any qualified scientist can request access to data by submitting a study proposal form.
Types of analyses: All data request proposal will be reviewed by The Data Access and Publications Committee(DAPC; Dr. Leigh Johnson, Chair).
Mechanisms of data availability: After approval of a proposal
